# Verification of surface‐guided radiation therapy (SGRT) alignment for proton breast and chest wall patients by comparison to CT‐on‐rails and kV‐2D alignment

**DOI:** 10.1002/acm2.14263

**Published:** 2024-01-24

**Authors:** Hui Zhao, Vikren Sarkar, Sara St James, Adam Paxton, Fan‐Chi Frances Su, Ryan G. Price, Christian Dial, Matthew Poppe, David Gaffney, Bill Salter

**Affiliations:** ^1^ Radiation Oncology Department University of Utah Salt Lake City Utah USA; ^2^ University of Texas Southwestern Dallas Texas USA

**Keywords:** CTOR, kV‐2D, proton, SGRT

## Abstract

**Background:**

Surface‐guided radiation therapy (SGRT) systems have been widely installed and utilized on linear accelerators. However, the use of SGRT with proton therapy is still a newly developing field, and published reports are currently very limited.

**Purpose:**

To assess the clinical application and alignment agreement of SGRT with CT‐on‐rails (CTOR) and kV‐2D image‐guided radiation therapy (IGRT) for breast treatment using proton therapy.

**Methods:**

Four patients receiving breast or chest wall treatment with proton therapy were the subjects of this study. Patient #1′s IGRT modalities were a combination of kV‐2D and CTOR. CTOR was the only imaging modality for patients #2 and #3, and kV‐2D was the only imaging modality for patient #4. The patients’ respiratory motions were assessed using a 2‐min surface position recorded by the SGRT system during treatment. SGRT offsets reported after IGRT shifts were recorded for each fraction of treatment. The agreement between SGRT and either kV‐2D or CTOR was evaluated.

**Results:**

The respiratory motion amplitude was <4 mm in translation and <2.0^°^ in rotation for all patients. The mean and maximum amplitude of SGRT offsets after application of IGRT shifts were ≤(2.6 mm, 1.6^°^) and (6.8 mm, 4.5^°^) relative to kV‐2D‐based IGRT; ≤(3.0 mm, 2.6^°^) and (5.0 mm, 4.7^°^) relative to CTOR‐based IGRT without breast tissue inflammation. For patient #3, breast inflammation was observed for the last three fractions of treatment, and the maximum SGRT offsets post CTOR shifts were up to (14.0 mm, 5.2^°^).

**Conclusions:**

Due to the overall agreement between SGRT and IGRT within reasonable tolerance, SGRT has the potential to serve as a valuable auxiliary IGRT tool for proton breast treatment and may improve the efficiency of proton breast treatment.

## 1 INTRODUCTION

Surface‐guided radiation therapy (SGRT) has been routinely utilized for breast radiation therapy over the last decade due to its ease of use, stability, reproducibility, and accuracy on patient setup, target localization and intra‐fractional motion monitoring.^1^, ^2^, ^3^, ^4^

SGRT systems have been widely installed and utilized on linear accelerators, including all three commercially available SGRT systems mentioned in the report of AAPM TG302^1^: AlignRT (VisionRT, London, United Kingdom), Catalyst (C‐Rad, Sweden), and Identify (Varian Medical Systems, Palo Alto, CA). The use of SGRT with Proton therapy is still a newly developing field, and published reports are currently very limited.^5^, ^6^, ^7^ We believe this report to be the first verification of SGRT alignment accuracy compared to both daily CT‐on‐Rails (CTOR) and kV‐2D for breast proton treatment.

## 2 MATERIALS AND METHODS

**FIGURE 1:**
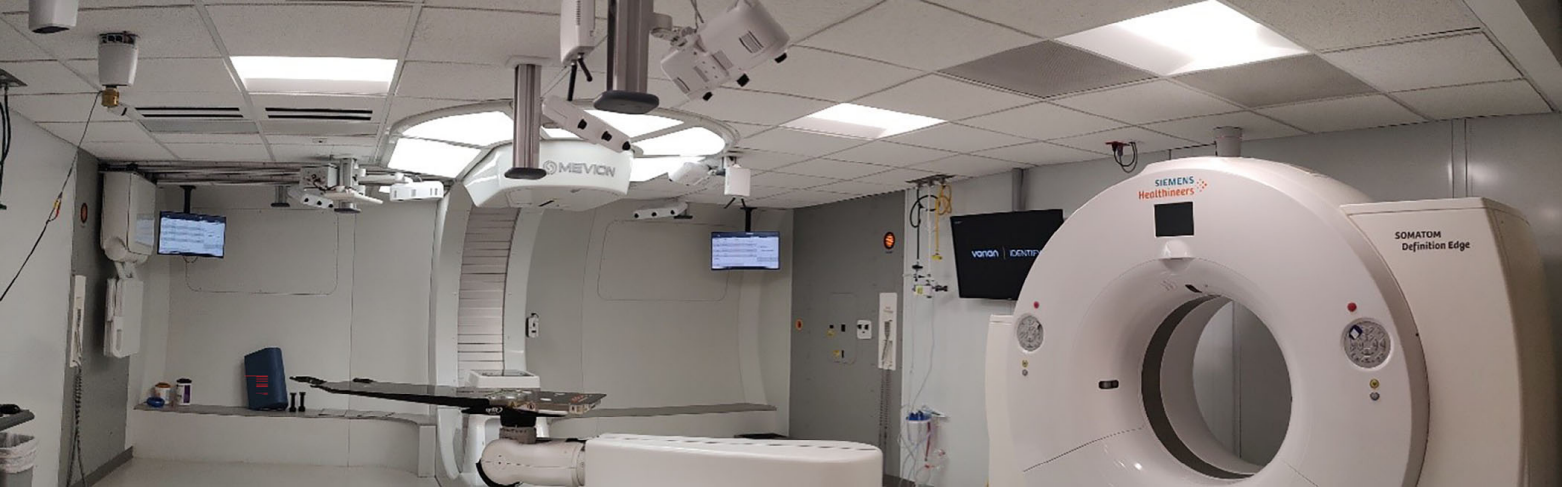
Proton vault at our center showing the half gantry proton delivery system, the CTOR system and Identify system.

**FIGURE 2:**
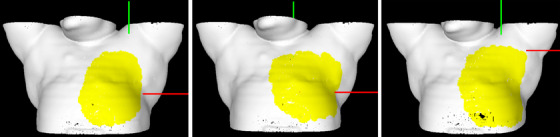
Identify ROIs for each treatment ISO for patient #2. The yellow area is the ROI.

Four patients receiving breast or chest wall treatment with proton therapy were the subjects of this study. Patient information is shown in Table 1, including patient age, treatment sites, prescription dose, fractionation, image guided radiation therapy (IGRT) modality, and the number of treatment plan isocenters (ISO). All four patient treatments were delivered using a Mevion s250i with HYPERSCAN (Mevion Medical Systems, Littleton, Massachusetts) proton system. The proton vault at our center is equipped with Mevion's kV‐2D planar imaging system (Verity), a Siemens Somatom Definition Edge 64 slice CTOR (Siemens Healthineers, Erlangen, Germany) and a Varian Identify SGRT research system (version 2.2) (Figure 1). After the treatment plans were created, the patient's surface contour and the treatment plan isocenter coordinates were exported from the treatment planning system (RayStation, version 11B, RaySearch Laboraties) to the Identify server. The region of interest (ROI) was drawn on the patient CT‐simulation DICOM surface using the Identify planning tool software. For patients #1, #3, and #4, the ROI included the entire treated breast, and for patient #2 (chest wall), each of the three ROIs included the relevant treatment areas. Figure 2 shows the three different ROIs for each treatment ISO for patient #2 (yellow area). The CT‐simulation DICOM surface was defined as the SGRT reference for the entire treatment course.

**TABLE 1:** Patient information.

Patient #	Age	Treatment sites	Rx dose & fractionation	IGRT modality	# ISO's
1	79	Left breast Lumpectomy boost	56 Gy/26fx	CTOR kV‐2D	1
2	49	Left chest wall Internal mammary lymph nodes Left axilla Supraclavicular lymph nodes	50 Gy/25fx	CTOR	3
3	56	Left breast Left supraclavicular lymph nodes	50 Gy/25fx	CTOR	2
4	68	Right breast Internal mammary lymph nodes Right axilla Right supraclavicular lymph nodes	42.56 Gy/16fx	kV‐2D	2

On the day of virtual simulation or treatment, the patient's respiratory motion was assessed to show the amplitude of patient surface intra‐fractional motion, by using a 2‐min recording of the surface position by the Identify SGRT system. For patient #1, during virtual simulation and the first six treatment fractions (0–6), both kV‐2D images and CTOR were acquired for IGRT while the remaining fractions (7–28) utilized only CTOR. A helical CTOR was acquired during patient's free breathing, using the energy of 120 kV, the same technique that was used for simulation CT. For kV‐2D images, posterior/anterior (PA) and left lateral orthogonal images were acquired. The energy of 75 kV was used for PA field and 100 kV was used for left lateral field. The field size of kV images were 32 cm × 32 cm (imager panel size 43 cm × 43 cm). The image registration for both CTOR and kV‐2D was based on soft tissue alignment. Initially, kV‐2D and CTOR were both used in order to evaluate the potential of kV‐2D alone for IGRT, as CTOR significantly increases patient time on the table. kV‐2D was acquired first, and the suggested shifts were applied. The verification CTOR was then acquired, and the suggested shifts were applied as final patient treatment position. For patient #2 and #3, only CTOR was utilized for IGRT. Patient #4 has chronic obstructive pulmonary disease (COPD) and it was difficult for her to remain still on the treatment table. Therefore, only kV‐2D was used as daily IGRT to shorten her time on the table. After IGRT shifts from either kV‐2D or CTOR were applied, Identify SGRT patient surface offset tracings were recorded for every treatment isocenter and for each treatment fraction. For each isocenter and every fraction, 20 second of SGRT offset tracing post IGRT shifts was averaged. This average value represents the surface displacement between the SGRT and IGRT and accounts for breathing motion. For patient #1, a set of post‐IGRT‐shift SGRT offsets was snapshotted to represent the displacement between the SGRT and IGRT. The reason of not using the averaged offset tracing was her negligible respiratory motion amplitude (≤1.2 mm). The mean and maximum amplitude of SGRT offsets for each isocenter and each patient over the entire treatment were calculated. The degree of agreement between SGRT and either kV‐2D or CTOR was evaluated.

## 3 RESULTS

Table 2 shows the respiratory motion amplitude recorded by the Identify system for all patients. All patient respiratory motion amplitudes within the 2‐min recording were <4 mm in translation and <2.0^°^ in rotation. The respiratory motion amplitude represents the patient surface intra‐fractional motion amplitude.

**TABLE 2:** Respiratory motion amplitude for all patients.

Patient #	Vert (mm)	Long (mm)	Lat (mm)	Yaw^0^	Roll^0^	Pitch^0^
1	1.2	1.2	0.7	1.0	0.9	0.8
2	2.6	2.8	3.2	1.9	0.8	1.4
3	1.2	1.5	1.6	0.8	0.5	0.6
4	2.3	3.9	1.2	0.9	0.6	0.8

Table 3 shows the mean and maximum amplitude of Identify‐recorded SGRT offsets post IGRT shifts for both kV‐2D and CTOR for all patients. For the virtual simulation and the first six treatment fractions, the mean SGRT offsets for patient #1 after application of IGRT shifts were (≤2.6 mm in translation, ≤1.6^°^ in rotation) relative to kV‐2D, and (≤1.7 mm, ≤1.2^°^) relative to CTOR. The maximum SGRT offsets post IGRT shifts were (6.8 mm, 3.2^°^) relative to kV‐2D, and (3.6 mm, 1.9^°^) relative to CTOR. The SGRT offsets post IGRT shifts were smaller for CTOR than for kV‐2D. For the entire course of treatment, the mean SGRT offsets post CTOR shifts were (≤1.6 mm, ≤1.2^°^), and the maximum SRGT offsets were (4.4 mm, 3.5^°^). The mean SGRT offsets of all three isocenters for patient #2 post CTOR IGRT shifts were (≤2.0 mm, ≤1.1^°^), and the maximum SGRT offsets were (4.9 mm, 2.8^°^). The mean SGRT offsets of both ISOs for patient #3 post CTOR IGRT shifts were (≤5.2 mm, ≤2.7^°^). Breast inflammation was observed for the last three‐fractions of treatment, and the mean SGRT offsets without the last three‐fractions for both isocenters were (≤3.0 mm, ≤2.0^°^). The maximum SGRT offsets were (14 mm, 5.2^°^) for the entire treatment, and (3.9 mm, 2.6^°^) without the last three‐fractions. The mean SGRT offsets of both ISOs for patient #4 post kV‐2D IGRT shifts were (≤2.8 mm, 2.6^°^), and the maximum SGRT offsets were (5.0 mm, 4.7^°^).

**TABLE 3:** SGRT offsets post kV‐2D and CTOR IGRT shifts for all patients. In the mean columns, the mean ± standard deviation are listed.

		SGRT offsets post IGRT shifts for patient #1					
		Vert (mm)	Long (mm)	Lat (mm)	Yaw^0^	Roll^0^	Pitch^0^
Mean	kV‐2D (0‐6)	2.2 ± 1.7	2.6 ± 2.0	1.3 ± 1.0	1.6 ± 1.0	0.5 ± 0.4	0.6 ± 0.5
	CTOR (0‐6)	1.7 ± 1.2	1.1 ± 0.8	0.9 ± 1.1	1.2 ± 0.7	0.7 ± 0.3	0.4 ± 0.4
	CTOR (0‐28)	1.6 ± 1.2	1.2 ± 1.0	1.5 ± 1.2	0.8 ± 0.6	1.2 ± 0.8	0.5 ± 0.6
Max	kV‐2D (0‐6)	5.0	6.8	3.0	3.2	1.1	1.6
	CTOR (0‐6)	3.6	2.2	3.1	1.9	1.2	1.2
	CTOR (0‐28)	4.3	3.5	4.4	1.9	3.5	2.9

	Iso #	SGRT offsets post CTOR shifts for patient #2					
		Vert (mm)	Long (mm)	Lat (mm)	Yaw^0^	Roll^0^	Pitch^0^
Mean	Iso1	1.2 ± 0.9	2.0 ± 1.3	1.5 ± 1.3	0.6 ± 0.3	0.6 ± 0.5	0.6 ± 0.6
	Iso2	1.4 ± 1.0	1.4 ± 1.0	1.7 ± 0.9	1.0 ± 0.7	0.9 ± 0.8	0.6 ± 0.5
	Iso3	1.1 ± 0.6	1.6 ± 1.2	1.6 ± 1.0	0.8 ± 0.7	1.1 ± 0.7	0.6 ± 0.4
Max	Iso1	2.6	4.9	4.7	1.3	1.8	2.6
	Iso2	4.5	4.4	3.8	2.6	2.8	1.9
	Iso3	2.8	4.0	3.6	2.3	2.4	1.5

	Iso #	SGRT offsets post CTOR shifts for patient #3					
		Vert (mm)	Long (mm)	Lat (mm)	Yaw^0^	Roll^0^	Pitch^0^
Mean	Iso1	1.3 ± 1.0	1.2 ± 1.0	5.2 ± 4.0	2.5 ± 1.4	1.5 ± 1.6	0.4 ± 0.3
	Iso2	1.0 ± 0.8	1.1 ± 1.0	1.4 ± 1.3	2.7 ± 1.3	1.5 ± 1.5	0.6 ± 0.4
Max	Iso1	2.5	2.8	14.0	5.2	4.8	1.0
	Iso2	2.9	3.2	3.6	5.2	4.8	1.1

	Iso #	SGRT offsets post kV‐2D shifts for patient #4					
		Vert (mm)	Long (mm)	Lat (mm)	Yaw^0^	Roll^0^	Pitch^0^
Mean	Iso1	1.7 ± 1.2	2.4 ± 1.5	1.8 ± 1.7	0.3 ± 0.3	2.2 ± 1.0	0.4 ± 0.3
	Iso2	2.4 ± 1.5	2.8 ± 1.4	2.1 ± 1.4	1.0 ± 1.1	2.6 ± 1.3	0.9 ± 0.8
Max	Iso1	3.7	4.6	4.5	1.1	3.7	1.0
	Iso2	4.7	5.0	4.5	4.5	4.7	3.3

## 4 DISCUSSION

In this study, the small mean translational and rotational SGRT offsets post IGRT (CTOR and kV‐2D) shifts (≤3.0 mm, ≤2.6^°^) suggest general agreement between SGRT and IGRT on all proton breast and chest wall patient treatments without tissue inflammation. This result agrees with published investigations regarding SGRT compared to CTOR and SGRT compared to x‐ray radiograph for partial breast and chest wall irradiation.^7^, ^8^, ^9^ The most commonly used IGRT modality for photon breast and chest wall treatment are tangential field radiographs. Non‐tangential kV‐2D is a standard IGRT tool for bony anatomy alignment, but not an ideal IGRT modality for breast and chest wall treatment, due to its limited soft tissue contrast and inability to accurately assess rotational roll. CTOR provides the same image quality as simulation CT, and the image registration process is more straightforward than kV‐2D registration. However, it takes approximately five total minutes for the CTOR process, including proton couch movement from the patient setup position to CTOR position, performing the CTOR, registration of imaging and couch movement back to proton patient setup position. During this extended process there is potential for patient motion. In this study, we observed patient intra‐fractional motion occasionally reaching 7 mm (patient #2) in translation from setup to the end of CTOR via Identify intra‐fractional monitor function.

Our proton system uses a half gantry configuration, and a gantry‐mounted CBCT system is not possible. With an increasing number proton centers installing systems with a half gantry configuration, CTOR or CBCT using a separate system becomes a logical IGRT consideration. However, full volumetric imaging with such systems carries with it the overhead of an extended timeframe.

The agreement between SGRT and CTOR was within 4.9 mm and 3.5^°^ maximum for all four patients’ entire treatment courses in this study. Contributing factors to this difference surely include deformation‐effects in the image registration process between SGRT and CTOR, along with patient intra‐fractional motion between the time of CTOR and the time of SGRT offsets recording. Another contributing factor was the uncertainty of reference surface derived from free breathing simulation CT, which mixed the entire respiratory phases. The manual image registration was performed in this study for both CTOR and kV‐2D, based on soft tissue alignment in the treatment area. The Identify SGRT surface‐matching algorithm is based on rigid registration inside the ROI. The CTOR was considered as ground truth in this study. In this context, the 4.9 mm and 3.5^°^ maximum differences are reasonable; given the respiratory motion (intra‐fractional motion) for all patient were as much as 4 mm in translation and up to 2.0^°^ in rotation. Zhao et al. reported maximum displacement of ≥5 mm between SGRT and CTOR in nine out of 12 patients in their partial breast irradiation investigation.^8^ We note that the agreement between SGRT and CTOR is within the 5 mm robustness criterion used for all patients’ proton treatment plans. We believe that this degree of agreement is indicative of an acceptable degree of correlation between the patient's simulation‐derived surface and the internal anatomy that was used for CTOR alignment. And, therefore, that it is feasible to utilize daily SGRT as an auxiliary IGRT modality, combined with less frequent (once or twice weekly instead of daily) CTOR.

In this study, two treatment fields for each treatment isocenter were planned for all patients, one en face and one oblique beam with pencil beam scanning technique. Due to the 20 × 20 cm field size of our proton system, field matching was achieved with robust optimization and junctions between fields were evaluated during treatment planning for multi‐isocenter treatment. Only one set of kV‐2D and/or CTOR were acquired for IGRT for each fraction multi‐isocenter treatment. Since offsets along the beam direction have negligible effect on the delivered dose distribution for proton beams, the lateral and longitudinal offsets are most significant for en face beams, and all principal direction offsets are clinically significant for oblique beams.

**FIGURE 3:**
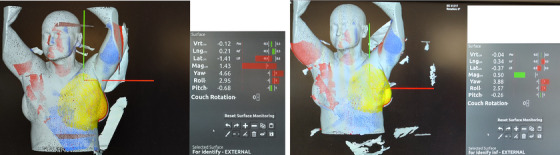
Identify surface matching for patient #3 (treatment fraction 25). The left screenshot is for ISO1, and the right is for ISO2.

Non‐trivial differences of SGRT offsets for two isocenters were observed on the last three‐fractions for patient #3 (Figure 3). The same ROI was used as reference for both isocenters. The lateral offset was 14.1 mm for ISO1, and 3.7 mm for ISO2. The most plausible causes of the discrepancy are the different locations of the patient inflamed breast relative to IGRT cameras and the impact of the inflammation on the rigid registration algorithm between ISO1 and ISO2. The breast inflammation was also apparent on CTOR, and the image registration was performed based on the chest wall alignment. The treatment plan was re‐calculated on the CTOR scan, and the dose distribution, target coverage and organ at risk dose were evaluated before the treatment delivery.

Due to the small number of patients in this study, the data was not sufficient to perform a statistical analysis. This is the limitation of the study.

Finally, we note that the real‐time continuous surface tracking function of SGRT can provide invaluable patient intra‐fractional motion monitoring throughout patient treatment.

## 5 CONCLUSIONS

Due to the overall agreement between SGRT and IGRT within reasonable tolerance, SGRT has the potential to serve as a valuable auxiliary IGRT tool for proton breast treatment and may improve the efficiency of proton breast treatment.

## AUTHOR CONTRIBUTION

Hui Zhao: First author, study design, manuscript drafting, intellectual content contribution; Vikren Sarkar: Direct intellectual content contribution; Sara St. James: Direct intellectual content contribution; Adam Paxton: Direct intellectual content contribution; Fan‐Chi Frances Su: Direct intellectual content contribution; Ryan G. Price: Direct intellectual content contribution; Christian Dial: Direct intellectual content contribution; Matthew Poppe: Direct intellectual content contribution; David Gaffney: Direct intellectual content contribution; Bill Salter: Direct intellectual content contribution.

## CONFLICT OF INTEREST STATEMENT

The authors declare no conflicts of interest.
